# Fishermen as a Suitable Population for HIV Intervention Trials

**DOI:** 10.1155/2010/865903

**Published:** 2010-07-18

**Authors:** Zachary A. Kwena, Craig R. Cohen, Norton M. Sang, Musa O. Ng'ayo, Jeremiah H. Ochieng, Elizabeth A. Bukusi

**Affiliations:** ^1^Research Care and Treatment Program, Center for Microbiology Research, Kenya Medical Research Institute, Lumumba Health Center, P.O. Box 614, 40100, Kisumu, Nairobi, Kenya; ^2^Department of Obstetrics, Gynecology and Reproductive Health, Bixby Center for Global Reproductive Health, University of California, San Francisco, CA 94105, USA

## Abstract

*Background*. Suitable populations to sustain continued evaluation of HIV and sexually transmitted infection (STI) prevention interventions are required. We sought to determine whether fishermen are a suitable population for HIV intervention trials. *Methods*. In a cross-sectional descriptive survey, we selected 250 fishermen from proportional to size sampled boats. We collected socioeconomic and behavioral information, and specimens for HIV, herpes simplex virus (HSV-2), syphilis, gonorrhea, chlamydia and human papillomavirus (HPV) tests from consenting participants. *Results*. One third of the fishermen had concurrent sexual partnerships and two thirds were involved in transactional sex. About 70% were involved in extramarital sex with only one quarter using condoms in their three most recent sexual encounters. HIV prevalence was 26% and HSV-2 and HPV was 57%. Over 98% were willing to participate in a future HIV prevention clinical trial. *Conclusion*. Fishermen are a high-risk group for HIV/STI infections that may be suitable for HIV prevention trials. A cohort study would be useful to measure the incidence of HIV/STIs to ultimately determine the feasibility of enrolling this population in an HIV/STI prevention clinical trial.

## 1. Introduction

There is urgent need to continue evaluating interventions such as microbicide product(s) that have potential to prevent transmission of sexually transmitted infections (STIs) including HIV. These evaluations require suitable populations that not only have high-risk sexual behaviors and STI/HIV incidence, but would also benefit and have interest in study participation [1–4].Issues of low-retention rates in randomized clinical trials hamper trial progress even in populations that have high-risk sexual behavior and HIV prevalence which results in inadequate statistical power to measure the effect of the intervention on the primary outcome, for example, HIV incidence [3].

Sufficiently high risk sexual behaviors that result in HIV acquisition in a population of interest [4, 5] cushions against the likely changes in behavior that result from “the trial effects” of counseling and treatment. These changes in sexual behavior may interfere with generating data that has adequate statistical power to measure the benefits of an intervention [6–9]. However, finding such populations that are suitable for HIV intervention trials remains a big challenge, even as new and more promising interventions emerge [6, 10, 11]. 

Fishermen in Kenya, like in many other parts of the world, are usually young and highly mobile, often staying away from their families for long periods in addition to interacting a lot with women who trade in fish [12–15]. This effectively puts them at increased risk of engaging in high-risk sexual behavior with concomitant negative outcome. We, therefore, conducted a cross-sectional descriptive survey to determine whether fishermen along Lake Victoria in Kisumu, Kenya would be a suitable population for an HIV prevention trial such as a topical male microbicide.

## 2. Methods

### 2.1. Design and Study Population

We conducted a cross-sectional descriptive survey along Lake Victoria in Kisumu between August and December 2005. In this survey, we selected participants from 18 of the 32 beaches using a proportional to size sampling method based on the number of registered boats.

Each beach had a primary and a secondary list of boats randomly selected from the registered boats at these beaches. The secondary list was only used in the event that a boat on the primary list was no longer in operation or participant(s) from selected boat(s) decline to participate or fail to meet inclusion criteria. If this happened, the boats from the secondary list were selected sequentially to replace those on the primary list. Ideally, four crewmembers from each boat were conveniently selected to participate in the study giving a sample of 250 participants. Kenya Medical Research Institute and University of Washington Ethical Review Committees approved the study before implementation and the study procedures were conducted with the understanding and the consent of the participants.

### 2.2. Measurements

#### 2.2.1. Survey Questionnaire and Specimen Collection

Data collection for the study was preceded by intense community mobilization and engagement using both local administration and beach management structures to prepare the community. This involved informing potential participants about the design of different phases of the study that included information on randomization, blinding, placebo and followup visits in their weekly assemblies organized by beach management officials. We also discussed various aspects of trial design with participants in focus group discussions that we conducted prior to survey.

During data collection, participants were consented and asked to respond to a standardized structured interview in private by a social scientist. We gathered information on their socio-economic and demographic details and also collected data on each of their last three sexual partners in respect to condom use, concurrent relationships and transactional and extramarital sex. We also asked them about their interest in participating in a future HIV/STI prevention trial—at this point only giving details of planned sample size and its duration, migration patterns, and length of time they will continue working in the fishing industry. Further, we provided pre- and post-test counseling for HIV and took blood samples for HIV-1, HSV-2, and syphilis serological tests; urine for gonorrhea and chlamydia testing and genital swabs for the identification of HPV. Appropriate treatment and/or referrals was given for those diagnosed with STIs.

#### 2.2.2. Laboratory Assays

Serum was divided for three sets of tests. HIV-1 serology was carried out as per the Kenyan Ministry of Health guidelines; first using determine HIV-1/2 (Abbott Laboratories, IL) and Uni-Gold HIV (Trinity Biotech PLC, Bray, Ireland) and the results confirmed using the p24 ELISA kit Vironostica, (Organo Tednika, Netherlands) to resolve any discrepancies between the first two assays. Syphilis infection was determined using Macro-Vue Rapid Plasma Reagin test (Benex limited, Ireland). HSV-2 infection was first determined using HerpeSelect HSV-2 ELISA (Focus Diagnostics, Cypress, CA) and confirmed by the University of Washington western blot assays [16]. *Chlamydia trachomatis* and *Neisseria gonorrhoeae* testing were done using the APTIMA Combo 2 assay (Gen-Probe, Inc., San Diego, CA). HPV positivity was determined first by polymerase chain reaction (PCR) amplification using HPV L1 consensus primers MY09/MY11/HMB01 and *β*-globin primers PC04/GH20, followed by dot blot hybridisation with generic HPV and b-globin probes [17]. All the assays procedures and results interpretations were done according to the manufacturer's instructions.

#### 2.2.3. Outcome Variable

The outcome variables for this study were related to the suitability of the population for a male microbicide trial. We used sexual behavior, STI/HIV prevalence, reported interest in participating in subsequent trials, and retention potential of the fishermen as a proxy to determine whether they were a suitable population or not. We defined a suitable population as one with high-risk sexual behavior (that expose them to the risk of infection), high prevalence of STI/HIV (that is significantly greater than the prevalence in the general population), and one that would be willing and available for follow up visits throughout the duration of a proposed intervention trial.

### 2.3. Data Analysis

The data was collected on Teleform-created case report forms and scanned into the Teleform software (Cardiff, Vista, CA) before exporting into SPSS (Version 13.0, SPSS Inc., Chicago, IL). We analyzed the data exclusively by descriptive statistics using frequencies, percentages, cross-tabulations, medians, and modes.

## 3. Results

### 3.1. Socioeconomic and Demographic Characteristics of the Study Population

Of the 250 fishermen who participated in the survey, half were over 26 years old (range: 18–63 years). About three quarters (182/250) had at least 8 years of basic education and slightly over two-thirds (174/250) were married, of which 14% were polygamous. A quarter of them (60/250) lived in a house with a single room. As would be expected, fishing was main source of income for almost all of them; their median monthly income was Kshs. 5500 (80 USD). While almost everyone had a functional radio 96% (176/184) only 10% (10/184) had a functional television (Table 1) (The total number of participants is less than 250 because it is a subpopulation, that is, those who were staying in their ancestrial homes at the time of the study). 

### 3.2. Sexual Behavior of the Fishermen

#### 3.2.1. Condom Use and Concurrent Sexual Relationships

About one third (37/124) of the fishermen reported always using condoms with their three most recent sexual partners (Table 2). Of the 91 fishermen whose most recent sexual encounter was with a nonspousal partner, slightly over one fifth used condoms. Even in cases where the fishermen suspected their partners to be involved in romantic relationship with other fishermen or to have an STI, condom use was still low (26% and 27%, resp.). Of 100 fishermen who provided dates of sexual liaisons, over one third were involved in concurrent sexual partnerships (Table 2), with 15% having more than one concurrent sexual relationship. Of those who had concurrent relationships, fewer than one fifth used condoms with all of their three most recent sexual partners.

#### 3.2.2. Transactional and Extramarital Sexual Relationships

Of the 250 fishermen who participated in the study, two thirds were involved in transactional sex and 14% had transactional sex with all of their three most recent sexual partners. Other than money that was used by most of the fishermen in exchange for sex (62%), shoes, clothes, and the right to buy fish was also used (38%). Out of 175 fishermen who were involved in transactional sex, approximately a quarter (28%) used condoms in all their three most recent sexual encounters. Of the 174 fishermen who were married, almost all were involved in extramarital sex of whom one quarter used condoms in all their three most recent sexual encounters. (The total number of participants is less than 250 because it is a sub-population, that is, those who were married at the time of the study.) (Table 2).

### 3.3. Prevalence of STI Infection

Out of the 250 fishermen who consented to study participation, slightly over one quarter were HIV positive and twice the number were HSV-2 and HPV positive. Ten percent were positive for syphilis, and *Chlamydia trachomatis* and* Neisseria gonorrhoeae* were rarely detected (Table 2). Four people (2%) were coinfected with HIV-1, syphilis, HPV, and HSV-2 while one person was infected with HIV-1, HPV, HSV-2, and *C. trachomatis *and another with HIV-1, HPV, HSV-2, and *N. gonorrhoeae*. About 42% of the fishermen were infected with oncogenic HPV types of which the prevalence of HPV-16 was 12%, HPV-35 and HPV-52 10%, and HPV-18 7%. The low-risk HPV types accounted for 43% of all the HPV infection detected. The prevalence of HPV-62 was 11%, HPV-6 10%, and HPV-70 8%.

### 3.4. Willingness and Ability to Participate in the Trial

Ninety eight percent of the fishermen (244/250) expressed willingness to participate in a clinical trial to determine the efficacy of a male microbicide to prevent HIV/STI. Two thirds of the fishermen (169/248) had worked at the same beach in the year preceding the study, with almost a half of them on the same boat (Table 2). About a third (42/139) reported that they planned to continue working in the fishing industry for at least the next six years. (The total number of participants is less than 250 because of the missing variables.) Of the 32% (79/169) who had worked at different beaches in the previous year, only one quarter worked at beaches outside of Kisumu District. 

## 4. Discussion

This study set to determine whether fishermen along Lake Victoria in Kisumu could be a suitable population for a topical male microbicide HIV/STI prevention trial. Our survey shows that fishermen may be a suitable population for a male microbicide trial as supported by their high-risk sexual behaviors, high HIV, and viral STI prevalence and willingness to participate in future efficacy trial. We also found that these fishermen largely work on the same beach and anticipate to continue working in the fishing industry beyond five years. These results give us a proxy measure of the extent to which fishermen along Lake Victoria may be a suitable population for male microbicide trial that supports feasibility of planned efficacy study.

This formative study provided an important opportunity to assess whether fishermen in Kisumu could be a suitable population for a HIV intervention trial. Conducting trials in suitable populations ensure that trial objectives are achieved within specified time hence significantly cutting down on cost. Suitable populations necessarily need to have the following key characteristics such as: (1) high-risk sexual behavior that result in sufficiently high-HIV/STI incidence; (2) willingness to participate in a trial, and (3) ability to be retained during followup in the trial. Finding a population with these characteristics help to guarantee that sufficient outcome data are generated within a specified timeframe to measure the effect of the intervention [18, 19].

High-risk sexual behavior that is often associated with STI/HIV infections [20, 21] make fishermen a vulnerable population. Similar to our findings, other studies [13, 22, 23] have reported high-risk sexual behavior among fishermen fueled by the presence women fishmongers at fishing landing beaches [14]. Clearly, the nearly two-fold higher HIV prevalence found among these fishermen in comparison to the general male population in Nyanza suggests that the incidence of HIV is fueled by these high-risk sexual behaviors, such as lack of condom use and high frequency of concurrent, extramarital, and transactional sex [24–26].

Coupled with high-risk sexual behavior and STI/HIV prevalence, fishermen also reported high interest in participating in subsequent male microbicide trials. Some researchers have questioned the use of willingness to participate in studies obtained from formative surveys as a proxy measure of people's ultimate participation in the main trials [4]. This skepticism is based on the differences that have been observed between hypothetical question asked during formative surveys and actual participation in trials. Commonly, very limited information about the hypothetical clinical trial is given during the formative study about study participants, thus making it difficult for participants in the formative study to make an informed decision about the willingness to join a future study [27].

Prior to the beginning our data collection, we provided potential participants interviewers detailed information about the randomization process, use of a placebo, blinding, and the number and length of follow-up visits in their weekly assemblies organized by beach management officials. The same information was shared during focus group discussions. In the survey, we presumed that the participants would use this information that was now available in the community to respond to the question regarding willingness to participate in a future prevention trial. However, it is possible that some of the participants were new immigrants into the area or could not clearly recall the information. Other formative surveys [28, 29] report same kind of interest in target populations but what we need to measure is whether this hypothetical interest corresponds with actual interest in future prevention trials. 

Our findings depicted no apparent problem with the apparent mobility of the fishermen on future retention since their movement normally is between the beaches within Kisumu District. Thus, most fishermen in this population would be within a short commute to a study clinic, and would also be relatively nearby for tracing in case of missed appointments. However, due to their busy daily schedules, timings for the clinic follow-up visits and the actual amount time they spend at the clinic would need to be worked out carefully. 

Our study had a number of limitations that may constrain the interpretation of the results. First, the study did not measure HIV/STI incidence that could have been a better measure of suitability of fishermen for a topical male microbicide but instead used HIV/STI prevalence. In addition, as a cross-sectional study, we could not accurately assess retention. A future cohort study to measure incidence of HIV/STI would enable an accurate measure of both, and therefore would be a better assessment of whether fishermen in Kisumu would be suitable for an HIV/STI prevention trial. Second, responses to questions delivered through a face-to-face interview for sensitive issues may be subject to social desirability bias resulting to either under- or overreporting especially on the matters of sexual behavior. In addition, asking questions that require participants to respond about prior behavior may be subject to recall bias. As such, our results should be seen as a first step to determine the suitability of this population for an HIV/STI prevention trial, and likely are not generalizable to other fishing populations. Thirdly, although boats were randomly selected, individual participants were conveniently chosen thereby potentially introducing bias in the data and limiting the generalisability of resultant findings. Despite these limitations that are common with cross-sectional formative surveys, this study has potential to shade light on the suitability of fishermen for HIV prevention clinical trials.

Judging from the high-risk sexual behavior, high-HIV/STI prevalence, and interest in participating in future prevention trials, fishermen on the beaches along Lake Victoria in Kisumu may be a suitable population for microbicide trial. Further evaluation of this population should include measurement of HIV/STI incidence and retention over an extended period of time.

## Figures and Tables

**Table 1:** Characteristics of 250 fishermen recruited in a preclinical survey to determine their suitability for a male microbicide trial.

Characteristic (*n* = 250)	Frequency (%)
Married	174 (70%)
Basic education (8 years)	182 (73%)
Profess catholic faith	71 (28%)
Stay in single-roomed house	60 (24%)
Have a functional radio—those staying at ancestral homes (*n* = 184)*	176 (96%)
Have a functional television—those staying at ancestral homes (*n* = 184)*	19 (10%)
Own fishing boat	70 (28%)
Own fishing net	102 (41%)
Own a mobile phone	34 (14%)

	Median (Mode)
Age (years)	26 (21)
Monthly income (Kenya shillings)	5500 (3000)
Number of children	2 (0)

*n < 250 due to use of sub-population.

**Table 2:** Factors depicting fishermen along Lake Victoria in Kisumu as a suitable population for a male microbicide trial.

Factor	Frequency (%)
*Sexual behavior *(*n* = *variable*)*	
Engage in extra-marital sex (for married men) (*n* = 174)**	170 (98%)
Involved concurrent relationship among the last three partners (*n* = 100)	38 (38%)
Transactional sex with at least one of the last three sexual partners (*n* = 175)	113 (65%)
Use condoms consistently with the last three sexual partners (*n* = 124)	37 (30%)

*STI/HIV prevalence *(*n* = 250)	
HIV-1	64 (26%)
HSV-2	185 (74%)
HPV	142 (57%)
Syphilis	24 (10%)
Gonorrhea and Chlamydia	11 (4%)
Non-specific urethritis	10 (4%)

*Interest and retention in trial *(*n* = *variable*)*	
Willing to participate in a 2-year efficacy trial (*n* = 250)	244 (98%)
Worked on only one boat in the year preceding the survey (*n* = 246)	111 (45%)
Worked on only one beach in the year preceding the survey (*n* = 248)	169 (68%)
Worked on a beach outside the district in the year preceding survey (*n* = 246)	64 (26%)
Will continue working in the fishing industry beyond five years (*n* = 139)	42 (30%)

*n < 250 due to missing variables; **n < 250 due to use of sub-population.

## References

[1] Fleming TR, Richardson BA (2004). Some design issues in trials of microbicides for the prevention of HIV infection. *The Journal of Infectious Diseases*.

[2] Schechter M (2002). HIV vaccine evaluation center in Rio de Janeiro, Brazil. *Vaccine*.

[3] Seage GR, Holte SE, Metzger D (2001). Are US populations appropriate for trials of human immunodeficiency virus vaccine? The HIVNET vaccine preparedness study. *American Journal of Epidemiology*.

[4] Schechter M, Do Lago RF, De Melo MF (2000). Identification of a high-risk heterosexual population for HIV prevention trials in Rio de Janeiro, Brazil. *Journal of Acquired Immune Deficiency Syndromes*.

[5] Rida W, Fast P, Hoff R, Fleming T (1997). Intermediate-size trials for the evaluation of HIV vaccine candidates: a workshop summary. *Journal of Acquired Immune Deficiency Syndromes*.

[6] De Zoysa I, Elias CJ, Bentley ME (1998). Ethical challenges in efficacy trials of vaginal microbicides for HIV prevention. *American Journal of Public Health*.

[7] Mercer CH, Wellings K, Macdowall W (2006). First sexual partnerships-age differences and their significance: empirical evidence from the 2000 British National Survey of sexual attitudes and lifestyles (‘Natsal 2000’). *Journal of Adolescent Health*.

[8] Weber AE, Craib KJP, Chan K (2001). Sex trade involvement and rates of human immunodeficiency virus positivity among young gay and bisexual men. *International Journal of Epidemiology*.

[9] Hughes G, Catchpole M, Rogers PA (2000). Comparison of risk factors for four sexually transmitted infections: results from a study of attenders at three genitourinary medicine clinics in England. *Sexually Transmitted Infections*.

[10] Mantell JE, Myer L, Carballo-Diéguez A (2005). Microbicide acceptability research: current approaches and future directions. *Social Science and Medicine*.

[11] Gross M (2004). HIV topical microbicides: steer the ship or run aground. *American Journal of Public Health*.

[12] Kissling E, Allison EH, Seeley JA (2005). Fisherfolk are among groups most at risk of HIV: cross-country analysis of prevalence and numbers infected. *AIDS*.

[13] Allison EH, Seeley JA (2004). HIV and AIDS among fisherfolk: a threat to ’responsible fisheries’?. *Fish and Fisheries*.

[14] Markussen M Coping with uncertainty: women in the informal fish processing and marketing sectors of Lake Victoria.

[15] Lavreys L, Rakwar JP, Thompson ML (1999). Effect of circumcision on incidence of human immunodeficiency virus type 1 and other sexually transmitted diseases: a prospective cohort study of trucking company employees in Kenya. *The Journal of Infectious Diseases*.

[16] Ng’ayo MO, Bukusi E, Morrow RA (2008). Sexual and demographic determinants for herpes simplex virus type 2 among fishermen along Lake Victoria, Kenya. *Sexually Transmitted Infections*.

[17] Manos MM, Ting Y, Wright DK, Furth M, Greaves M (1989). The use of polymerase chain reaction amplification for the detection of genital human papillomavirus. *Molecular Diagnostics of Human Cancer, Cancer Cells 7*.

[18] Cohen J (1994). AIDS vaccine research. U.S. panel votes to delay real-world vaccine trials. *Science*.

[19] Steele FR (1995). Gp120: good, bad or indifferent?. *Nature Medicine*.

[20] Gorbach PM, Drumright LN, Holmes KK (2005). Discord, discordance, and concurrency: comparing individual and partnership-level analyses of new partnerships of young adults at risk of sexually transmitted infections. *Sexually Transmitted Diseases*.

[21] Wardlow H (2007). Men’s extramarital sexuality in rural Papua New Guinea. *American Journal of Public Health*.

[22] Merten S ‘Fish for sex’ exchange in the Kafue Flats: risky opportunities of rural women.

[23] Lampinen TM, Chan K, Remis RS (2005). Sexual risk behaviour of Canadian participants in the first efficacy trial of a preventive HIV-1 vaccine. *Canadian Medical Association Journal*.

[24] Cohen CR, Montandon M, Carrico AW (2009). Association of attitudes and beliefs towards antiretroviral therapy with HIV-seroprevalence in the general population of Kisumu, Kenya. *PLoS ONE*.

[25] Mehta SD, Moses S, Agot K (2008). Herpes simplex virus type 2 infection among young uncircumcised men in Kisumu, Kenya. *Sexually Transmitted Infections*.

[26] Mattson CL, Bailey RC, Agot K, Ndinya-Achola JO, Moses S (2007). A nested case-control study of sexual practices and risk factors for prevalent HIV-1 infection among young men in Kisumu, Kenya. *Sexually Transmitted Diseases*.

[27] Suhadev M, Nyamathi AM, Swaminathan S (2006). A pilot study on willingness to participate in future preventive HIV vaccine trials. *Indian Journal of Medical Research*.

[28] Koblin BA, Heagerty P, Sheon A (1998). Readiness of high-risk populations in the HIV network for prevention trials to participate in HIV vaccine efficacy trials in the United States. *AIDS*.

[29] Ruzagira E, Wandiembe S, Bufumbo L (2009). Willingness to participate in preventive HIV vaccine trials in a community-based cohort in south western Uganda. *Tropical Medicine and International Health*.

